# Impact of Sinapic Acid on Bovine Serum Albumin Thermal Stability

**DOI:** 10.3390/ijms25020936

**Published:** 2024-01-11

**Authors:** Aurica Precupas, Vlad Tudor Popa

**Affiliations:** “Ilie Murgulescu” Institute of Physical Chemistry, Romanian Academy, Splaiul Independentei 202, 060021 Bucharest, Romania; aprecupas@icf.ro

**Keywords:** sinapic acid, serum albumin, thermal stability, denaturation, aggregation

## Abstract

The thermal stability of bovine serum albumin (BSA) in Tris buffer, as well as the effect of sinapic acid (SA) on protein conformation were investigated via calorimetric (differential scanning microcalorimetry—μDSC), spectroscopic (dynamic light scattering—DLS; circular dichroism—CD), and molecular docking approaches. μDSC data revealed both the denaturation (endotherm) and aggregation (exotherm) of the protein, demonstrating the dual effect of SA on protein thermal stability. With an increase in ligand concentration, (i) protein denaturation shifts to a higher temperature (indicating native form stabilization), while (ii) the aggregation process shifts to a lower temperature (indicating enhanced reactivity of the denatured form). The stabilization effect of SA on the native structure of the protein was supported by CD results. High temperature (338 K) incubation induced protein unfolding and aggregation, and increasing the concentration of SA altered the size distribution of the protein population, as DLS measurements demonstrated. Complementary information offered by molecular docking allowed for the assessment of the ligand binding within the Sudlow’s site I of the protein. The deeper insight into the SA–BSA interaction offered by the present study may serve in the clarification of ligand pharmacokinetics and pharmacodynamics, thus opening paths for future research and therapeutic applications.

## 1. Introduction

Thermal denaturation of proteins is a key factor in biotechnological processes and pharmaceutical applications. The complex influence of various ligands on the proteins unfolding is far from being elucidated. The interaction of proteins with polyphenols may result in various changes in the physicochemical and functional features for both sides. Additional information concerning the effect of phenolic compounds on protein thermal stability is needed to control the functional properties of proteins.

**Figure 1:**
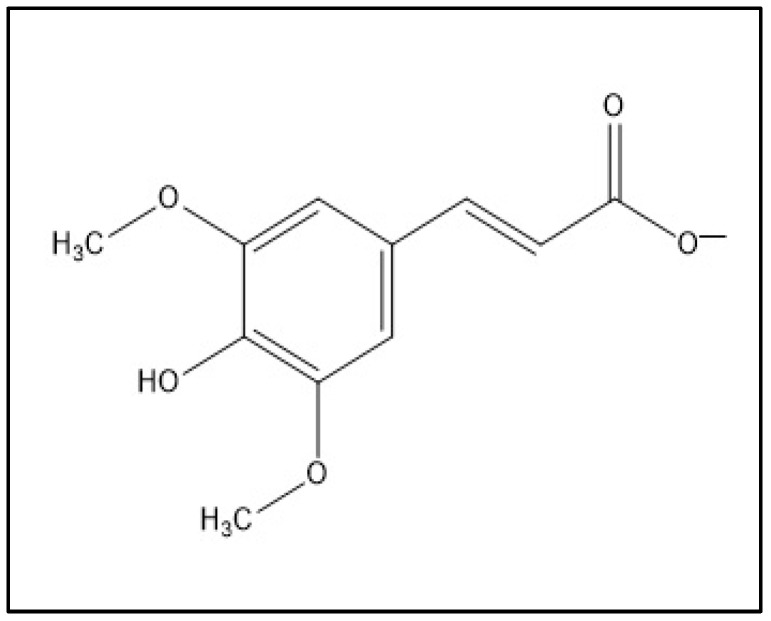
Chemical structure of carboxylate form of sinapic acid (SA).

Sinapic acid (SA, 3,5-dimethoxy-4-hydroxycinnamic acid) is a naturally occurring hydroxycinnamic acid, found in plants, fruits, vegetables, several cereals, and oil crops [1,2,3,4] with potential use in food processing, cosmetics, and in the pharmaceutical industry. SA acts as an anti-inflammatory [5], antidiabetic [6], anticancer agent [7] with antioxidant [8], antibacterial [9], chemopreventive [10], neuroprotective [11], and antihypertensive [12] effects. At pH 7.4, SA is present as an anion (Figure 1), the COOH group (pKa = 4.47) being dissociated [13].

Bovine serum albumin (BSA) is a globular protein that binds and transports a wide range of endogenous and exogenous ligands, playing a significant role in the pharmacokinetic behavior of a variety of drugs [14]. Its structural and functional similarities with the human serum albumin (HSA) made it a model protein for research purposes.

BSA presents two important binding sites for the ligands: Sudlow’s site I, located in subdomain IIA, and Sudlow’s site II, located in subdomain IIIA [15]. The native structure of BSA is the key to its biological function, but at high temperatures the protein loses its activity [16]. As a result, the thermal stability of protein is an active field of fundamental research, and the use of polyphenols to modulate aggregate formation seems very promising. The effect of SA on thermally driven denaturation and aggregation of serum albumin above its melting temperature is of great theoretical and practical importance since protein aggregation is associated with various diseases, such as Alzheimer’s and systemic amyloidosis [17]. The quest for new aggregation inhibitors for amyloid-forming proteins is crucial in the progress of novel therapeutics. Moreover, recent results pointed to thermal denaturation and aggregation as a threat to global distribution of biologics and research-use-only (RUO) proteins in a relatively high temperature environment [18]. Calorimetric [19], spectroscopic [20], and molecular docking [21,22] approaches are extensively used to study and analyze the interaction of different ligands with proteins and their applications in various research domains.

In our previous papers we evaluated the influence of different polyphenolic compounds on protein (BSA, hemoglobin) thermal stability. Furthermore, the aim of this study is to provide a better understanding of the impact of SA on heat-induced denaturation and aggregation of BSA. A previous study [23] using fluorescence measurements resulted in a binding constant of 4.5 × 10^5^ M^−1^ for SA to BSA at 298 K with a stoichiometry of 1.26. The site-marker competitive experiment indicated subdomain IIA of the protein as the binding site of SA. Accordingly, two concentrations of SA were used to evidence the impact of free (excess) and bound ligands on protein thermal stability. Also, the present study reveals the influence of Tris buffer on the protein aggregation process that could be calorimetrically evidenced.

## 2. Results and Discussions

### 2.1. Evaluation of Protein Thermal Stability by Differential Scanning Microcalorimetry (μDSC)

Previous calorimetric studies [24] concerning BSA thermal stability in phosphate buffer evidenced only the endothermal denaturation process. That was confirmed by our previous contributions [25,26,27]. In other words, the phosphate buffer covers the exothermal calorimetric signature of the aggregation process; although, the later takes place in the temperature range investigated. This is the reason for using complementary techniques to investigate the aggregation process in phosphate buffer: dynamic light scattering, asymmetric flow field-flow fractionation, and analytical ultracentrifugation [28]. Detection of both processes within the same DSC run was demonstrated by Barone et al. [24] in acetate buffer. The present study demonstrates the possibility of obtaining the DSC thermal signatures of protein denaturation and aggregation processes in Tris buffer. It also evidenced changes of these signatures produced by the presence of sinapic acid. Protein aggregation in Tris buffer is caused by the interaction of Tris buffer molecules with the exposed hydrophobic groups of denatured protein [29]. Tris buffer exhibits a pKa dependence with temperature [30]. However, pH measurements performed in the temperature range used for the μDSC method indicated that the results of this study were not significantly affected by the pH change with increasing temperature (Figure S1 from Supplementary Material).

**Figure 2:**
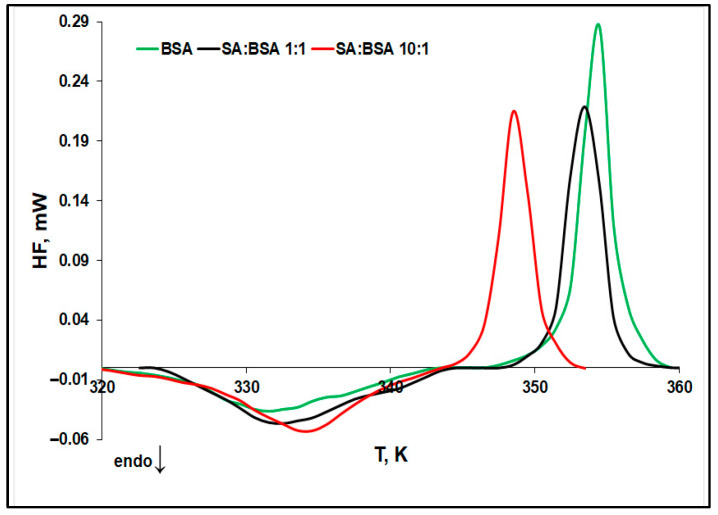
μDSC sigmoid baseline subtracted thermograms of BSA and BSA-SA systems.

Ligand binding on protein modifies the protein thermal stability, and this effect could be estimated by μDSC measurements [31]. Figure 2 presents the μDSC sigmoid baseline subtracted thermograms of the protein, in the absence and presence of SA, allowing for the evaluation of the ligand concentration influence on BSA thermal stability.

**Figure 3:**
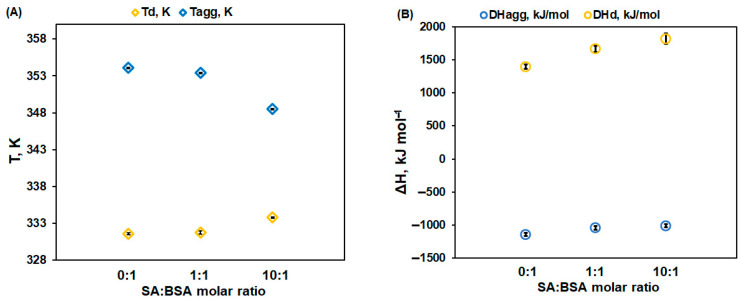
Thermodynamic parameters (**A**) temperatures and (**B**) enthalpies of BSA thermal denaturation and aggregation processes as a function of SA:BSA molar ratio. Standard deviations are presented as error bars.

Two signals could be noticed in the μDSC scans: (i) an endothermic one corresponding to the denaturation process of BSA, and (ii) an exothermic peak occurring at higher temperatures, related to the aggregation process of protein induced by the exposure of the hydrophobic residues. The thermodynamic parameters corresponding to the denaturation and aggregation processes of protein, in the absence and presence of increasing concentrations of SA, were calculated and are presented in Figure 3.

SA exerts a dual effect on BSA thermal stability in dynamic scanning conditions:

1. The denaturation temperature (T_d_) of protein increases with the SA concentration, pointing to a stabilization effect of SA on the protein native structure. Moreover, Figure 3 displays an increase in the denaturation enthalpy change (ΔH_d_) with an increasing in the ligand concentration. This may be ascribed to a lower exposure to the solvent molecules of the native protein’s buried hydrophobic regions, during the unfolding process in the presence of SA [32].
2. The aggregation temperature (T_agg_) and the absolute value of the enthalpy change (|ΔH_agg_|) decrease with SA concentrations: the ligand binding weakens the unfolded structure of protein. For a 10:1 SA:BSA molar ratio, the denaturation process and the aggregation one slightly overlap, producing a supplementary decrease in the calculated |ΔH_agg_| value.

SA binding to the native form of the protein delays the protein denaturation, manifesting as an increase in T_d_. A significant impact was observed for a 10:1 SA:BSA molar ratio, pointing to the stabilization effect of the excess free ligand. A similar influence was obtained for morin, a polyphenol that belongs to the flavanol class of flavonoids, in interaction with BSA [25]. The stabilization of protein structure could be generated by the electrostatic attraction between the negatively charged SA and the positively charged amino acid residues from the protein binding site, as observed by our group for the caffeic acid interaction with BSA [26].

The exposure of the hydrophobic core of the protein to the solvent molecules during unfolding is reduced in the presence of SA, the protein structure becomes more compact, and the ΔH_d_ value increases, as reported for the gallic acid effect on the BSA structure [27]. SA stabilizes the protein structure against thermal denaturation, in good agreement with data obtained by our group that report the enhancement of serum albumin thermal stability in the presence of various polyphenols (morin, caffeic acid, gallic acid, quercetin). The stabilization of the human serum albumin by the methotrexate drug [33], warfarin, and benoxaprofen [34] was also reported.

With increasing temperature, the hydrogen bonds that stabilize the native protein structure weaken, easing the exposure of hydrophobic protein groups to the solvent. As previously presented in the literature [29], a possible mechanism for the aggregation of BSA in Tris buffer is related to the favorable (exothermic) interaction of the hydrophobic groups of the unfolded protein with the buffer molecules. This favorable effect is added to the favorable protein–protein interaction and the result (the thermal fingerprint) is a well evidenced exothermic peak. As ligand binding to the unfolded/partially folded BSA decreases the T_agg_ and |ΔH_agg_| of the protein, SA acts as promoter of aggregation above the denaturation temperature. Also, the adsorption of free polyphenol on the protein surface could cause conformational changes in the protein structure that further promote aggregation [35]. A similar action was reported for morin binding on bovine serum albumin.

### 2.2. Circular Dichroism (CD) Spectra

**Figure 4:**
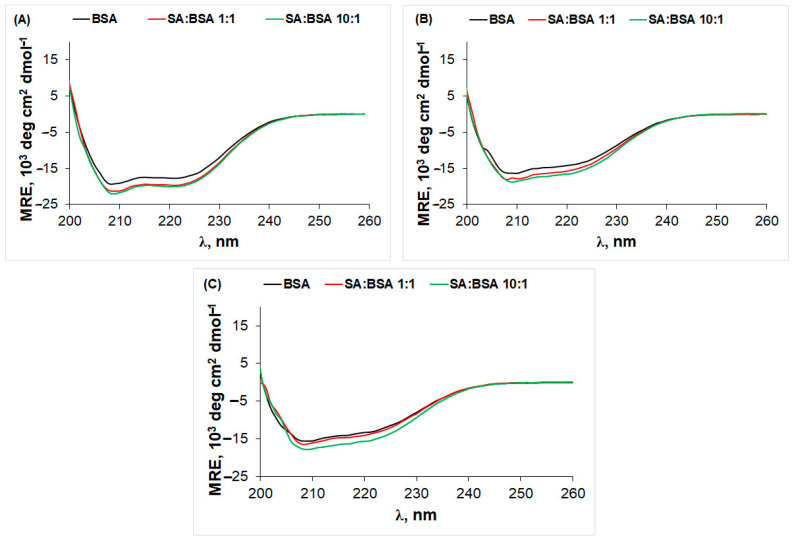
CD spectra of BSA in the absence and presence of different concentrations of SA after incubation at (**A**) 24 h 277 K, (**B**) 20 h at 338 K, and (**C**) 48 h at 338 K.

The conformational change in the protein’s secondary structure in static conditions (isothermal incubation) was monitored by CD. The influence of SA concentration on the BSA secondary structure after incubation at 277 K and 338 K is presented in Figure 4.

At 298 K, the protein secondary structure is predominantly α-helix, and SA presence induces an increase in the negative mean residue ellipticity (MRE) value, as a result of the ligand–protein interaction. The stabilization effect on the secondary structure of the protein, observed for both 1:1 and 10:1 SA:BSA molar ratios, could be correlated with μDSC data, where T_d_ increases in the presence of the ligand. Similar results were obtained for the interaction of morin [25], caffeic acid [26], and caffeoylquinic acids [36] with BSA.

After protein incubation at 338 K for 20 and 48 h, the CD spectra of BSA in the presence and absence of SA show a significant decrease in MRE values at 208 and 222 nm assigned to the α-helix structure, thereby indicating the thermal denaturation of the protein. Increasing the concentration of SA induces an increase in the CD signal even after incubation at 338 K. This is more evident after 48 h for a 10:1 SA:BSA molar ratio, pointing to the stabilization effect of the ligand on the protein structure. Both the bound ligand (1:1 SA:BSA molar ratio) and the free ligand (10:1 SA:BSA molar ratio) cause conformational changes in the protein structure.

**Figure 5:**
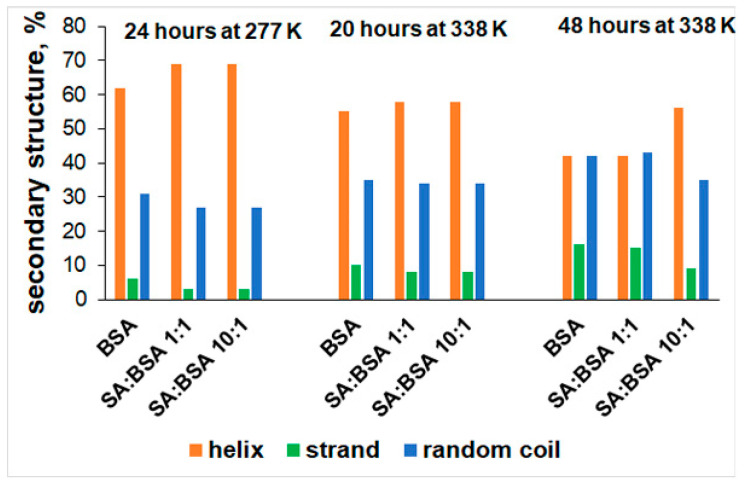
The secondary structure content of BSA and BSA–SA systems after incubation at 277 K and 338 K.

The protein secondary structure content obtained by Dichroweb (Figure 5) displays an increase in the α-helix structure with a simultaneous strand decrease for BSA in the presence of SA; this may be interpreted as complementary structural proof for the stabilization effect of SA evidenced in μDSC measurements.

After incubation at 338 K for 20 h, the α-helix content of BSA decreases, while the β and unordered structure increase, indicating the protein thermal denaturation. Increasing the concentration of SA stabilizes the protein structure, the α-helix content increases, and the strand and random coil contents slightly decrease. After 48 h at 338 K, the denaturation of protein is more evident, the native structure of the protein is disrupted along with a concomitant increase in the strand and random coil contents [37]. SA acts as a stabilizer only at high concentrations, enhancing the α-helix content, while the strand and random coil contents decrease.

### 2.3. Changes in the Hydrodynamic Dimeter of BSA Aggregates Investigated by Dynamic Light Scattering (DLS)

**Figure 6:**
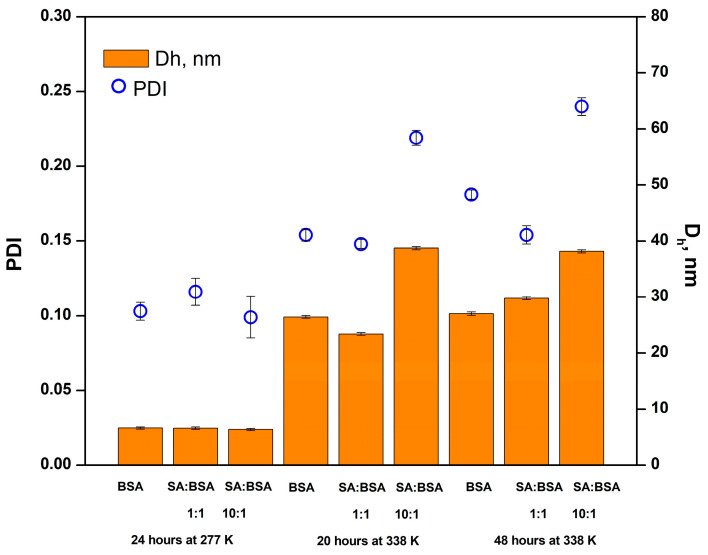
Hydrodynamic diameter and PDI of BSA population as a function of SA:BSA molar ratio. Standard deviations are presented as error bars.

DLS measurements were carried out to determine the sizes of aggregates formed after incubating the protein samples in the absence and presence of SA. The intensity and number size distributions (Figures S2 and S3 from Supplementary Material) of the BSA solution at 277 K show a single population with the hydrodynamic diameter (D_h_) value of 6.62 nm, corresponding to the native protein structure (monomeric form) which is in good agreement with the size of BSA reported in the literature [38]. In the presence of increasing concentrations of SA, no significant change in the D_h_ and polydispersity index (PDI) values could be observed (Figure 6).

After sample incubation for 20 h at 338 K, the DLS analysis still indicates a monomodal size distribution for the protein in the absence and presence of SA, with increasing particle size and PDI values as a consequence of denaturation and concomitant aggregation [39]. A high concentration of SA (10:1 SA:BSA molar ratio) induces a significant enhancement of PDI and D_h_ values, demonstrating the free ligand impact on protein aggregation at high temperatures. For a low concentration of SA, the aggregation effect is reduced compared to free BSA. In time, at 338 K, the size distributions of BSA and BSA–SA systems slightly change, so that after 48 h, a single population corresponding to larger aggregates is evidenced by DLS measurements. With increasing the incubating time at 338 K, the PDI values increase, suggesting the presence of a larger size species as result of the aggregation effect of the ligand. The influence of SA on the size distribution of the protein population at high temperature is manifested in two ways: At a low concentration (90.9 μM), after 20 h at 338 K, SA binding stabilizes the protein structure, in agreement with the CD results; after a longer incubating time (48 h), the ligand binding causes an opposite effect, promoting aggregation. At a high concentration (909 μM), SA promotes protein aggregation after 20 h at 338 K and the effect is maintained in time at 48 h incubation, with the same intensity, in good agreement with the CD results.

### 2.4. Molecular Docking

**Figure 7:**
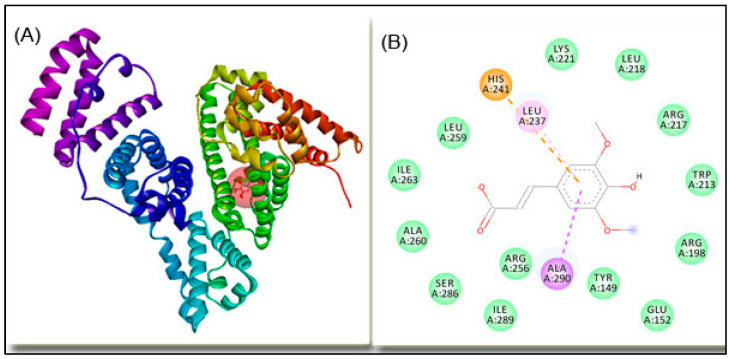
Molecular docking of SA and BSA; (**A**) SA is presented as stick and balls and the protein as solid ribbon. (**B**) The 2D representation of SA–BSA complex; the close amino acid residues are presented in green, dashed lines represent intermolecular interactions of different origin (electrostatic—orange line; π-alkyl—pink lines; π-σ—purple lines; π-π T-shaped—magenta lines; van der Waals interactions—light green lines).

Molecular docking of SA with BSA (Figure 7) was used to identify the most favorable binding site of the ligand within the native structure of the macromolecule and to find the amino acid residues involved in the interaction.

SA binds to subdomain IIA (Sudlow’s site I) of BSA with a binding affinity of −6.2 kcal/mol, corresponding to a binding constant of 3.52 × 10^4^ M^−1^, and the interaction is mainly driven by hydrophobic forces and electrostatic interaction, as shown in Table 1.

The present findings are consistent with the outcomes of previous investigations that indicated the binding of other polyphenols in the vicinity of TRP 213 of BSA [40,41].

## 3. Materials and Methods

### 3.1. Materials

BSA (A7030, lyophilized powder, purity ≥ 98%, CAS: 9048-46-8, Sigma-Aldrich, Saint Louis, MI, USA) and SA (D7927, purity 98.0%, CAS: 530-59-6, Sigma-Aldrich, Bangalore, India) were used with no further purification. Solutions of the protein and ligand were prepared in 25 mM Tris buffer (Trizma base, Honeywell Fluka, Charlotte, NC, USA, purity 99%) at pH 7.4. For μDSC, the samples of BSA (90.9 μM) in the absence and presence of SA were allowed to equilibrate for 24 h at 277 K and brought to room temperature before measurements. Similarly, for CD and DLS measurements, the protein samples (75 μM) in 25 mM Tris buffer, pH 7.4, were incubated at 277 K for 24 h in the absence and presence of ligand in various concentrations (SA:BSA molar ratio 1:1, 10:1). After this, the BSA samples were further used in different ways: (i) diluted to the working concentration (50 μM for CD and 37.8 μM for DLS measurements) and measured at 298 K, or (ii) kept at 338 K in an incubator without stirring. At certain time intervals (20 h and 48 h), aliquots were taken from solution, cooled to room temperature, diluted to the working concentration (50 μM for CD and 37.8 μM for DLS measurements), and used for measurements at 298 K.

### 3.2. Methods

#### 3.2.1. Differential Scanning Microcalorimetry (μDSC)

The thermal stability of BSA in the absence and presence of different concentrations of SA was studied in Tris buffer 25 mM, pH 7.4, using μDSC7 evo calorimeter (Setaram, Caluire, France) in the temperature range of 298 K–368 K, at 1 K min^−1^ heating rate. The thermodynamic parameters (enthalpy change and temperature) of denaturation and aggregation processes were determined by Calisto v.1077 software using a tangential sigmoid baseline. The per mol quantities were calculated from the absolute values (Joules), taking into account the sample volume and the protein concentration.

#### 3.2.2. Circular Dichroism (CD)

The secondary structure of protein and the effect of SA binding at 277 K and after incubation at 338 K were evaluated using CD measurements by a JASCO J-815 spectropolarimeter (Jasco, Japan), equipped with a Peltier-type temperature controller. Far-UV CD spectra of BSA were obtained with three accumulations for each measurement, using a 1 cm quartz cuvette. The protein concentration (0.5 μM) was maintained constant. The time constant, scan speed, resolution, and sensitivity were set at 1 s, 100 nm min^−1^, 1.0 nm, and 100 mdeg, respectively. The CD spectra of the samples were baseline-subtracted by using the spectrum of 25 mM Tris buffer, pH 7.4. The CD results were expressed in terms of mean residue ellipticity (MRE) in deg cm^2^ dmol^−1^ [36]. The secondary structure content of protein in the absence and presence of SA was evaluated using the K2D analysis algorithm [42] from the Dichroweb website [43] between 200 and 260 nm in order to remove the effect of chloride ions absorption below 200 nm [44]. Normalized root-mean square deviations (NRMSD) lower than 0.2 (the acceptable upper limit) were obtained for all fits of CD spectra [45,46] and are presented in Table S1 in Supplementary Material.

#### 3.2.3. Dynamic Light Scattering (DLS)

The hydrodynamic diameter (D_h_) of BSA (37.8 μM) and BSA–SA systems was determined by DLS measurements using a Nano ZS (Malvern Instruments, Worcestershire, UK) instrument with laser incident beam at λ = 633 nm and a fixed scattering angle of 173° equipped with a temperature-controlled system. A 0.22 μm pore-sized microfilter was used to filter all of the solutions. The analysis of intensity fluctuations enables the determination of the diffusion coefficients of particles, which are converted into a size distribution. Scattering data were collected as an average of 5 measurements [47], and the mean D_h_ value in the intensity distribution and the polydispersity index (PDI) were presented.

#### 3.2.4. Molecular Docking

Molecular docking study of SA binding with BSA was performed for predicting the binding site, the interaction forces, and the binding affinity. The protein structure (PDB ID: 4F5S) [48] was obtained from the RSCB Protein Data Bank [49]. The geometry of the SA anion was optimized by DFT/B3LYP/6-311G++ level of theory using the Gaussian 03 software [50], as previously described [51]. The molecular docking was performed using the Autodock Vina 1.1.2 software [52]. Autodock tools [53] were used for protein and ligand file preparation to add all hydrogen atoms, to assign the Gasteiger charges, to detect and assign the rotatable bonds of SA. The Lamarckian genetic algorithm was applied to determine the optimum binding site of the SA anion to BSA, set as rigid. The grid box which covers the amino acid residues of Sudlow’s site I and Sudlow’s site II was set up with 40 points in each of the X, Y, and Z dimensions centered on x, y, and z coordinates of 1 × 19 × 109, with a grid point spacing of 1 Å. The exhaustiveness of the global search was set to 8. The maximum number of binding modes was 9. The maximum energy difference between modes was 3 kcal/mol. The best docking mode in Autodock Vina was the minimum energy conformation of the ligand–protein complex (the largest ligand binding affinity). One possible (best) binding site was identified as Sudlow’s site I. The conformer with the lowest binding energy was selected for analysis. The type of interaction was evaluated using the BIOVIA Discovery studio 2019 [54].

## 4. Conclusions

The results of this study provide significant insight into polyphenol–protein interaction and its impact on protein thermal stability. The thermal stability of BSA and the impact of SA concentration were evaluated using calorimetric and spectroscopic approaches. μDSC data pointed to the dual action of SA on BSA thermal stability. On the one hand, SA binding to the native form of the protein increases its thermal stability, and on the other hand, SA promotes protein aggregation above the denaturation temperature. At a low concentration, bound SA interacts directly with BSA in a site-specific manner: the native structure of protein is stabilized, resulting in a reduced exposure of the partially unfolded protein surface upon heating. At higher concentrations, non-bound (free) SA present in excess may interact with the protein surface, with displacement of the solvent molecules and thereby screening the solvent–protein interaction and enhancing the thermal stability of the later [55]. Presented CD data revealed both conformational changes induced by SA binding that stabilizes the protein structure, and the protein partial unfolding after incubation at higher temperature. After heating above the denaturation temperature, aggregation of the partially unfolded molecules is possible. As evidenced by DLS measurements, the BSA aggregates increased in size with the increasing concentration of SA. Sudlow’s site I in subdomain IIA of BSA was identified as the binding site for the SA using molecular docking.

## Figures and Tables

**Table 1:** Molecular interactions between SA and the amino acid residues in BSA binding site obtained by molecular docking.

Amino acid Residue	Distance, Å	Type of Interaction
HIS241	4.75	electrostatic
ALA290	3.69	hydrophobic
HIS241	5.50	hydrophobic
LEU237	4.80	hydrophobic

## Data Availability

Data are contained within the article and the Supplementary Material.

## Supplementary Materials

The following supporting information can be downloaded at: https://www.mdpi.com/article/10.3390/ijms25020936/s1.
