# Supplementary material for: Impact of Sinapic Acid on Bovine Serum Albumin Thermal Stability
Source: Int J Mol Sci. 2024 Jan 11;25(2):936. doi: 10.3390/ijms25020936 (PMC10815719; doi:10.3390/ijms25020936)
Supplement: Supplementary file 1 [file ijms-25-00936-s001.zip › ijms-2801089-supplementary.pdf]

Supplementary Material

Impact of sinapic acid on bovine serum albumin thermal stability

Aurica Precupas and Vlad Tudor Popa*

"Ilie Murgulescu" Institute of Physical Chemistry of the Romanian Academy,
202 Splaiul Independentei, Bucharest 060021, Romania

***** Correspondence: vtpopa@icf.ro

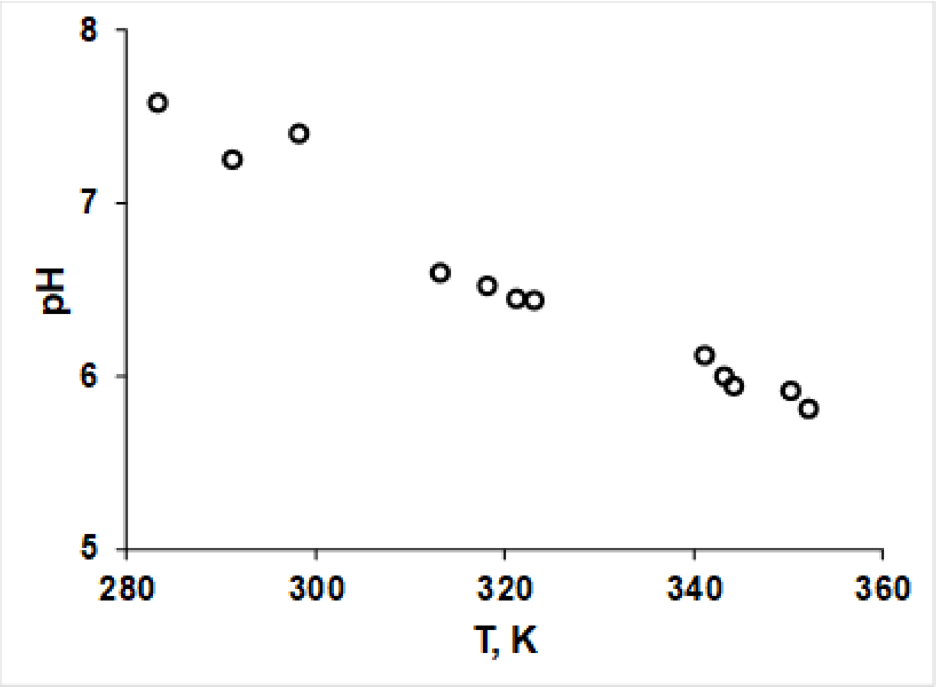

Figure S1. pH change with temperature for BSA solution in Tris buffer.

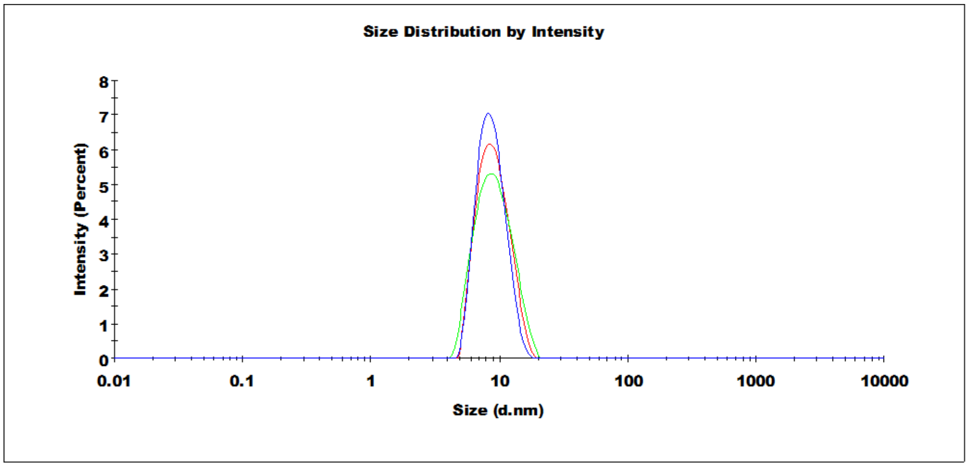

(A)

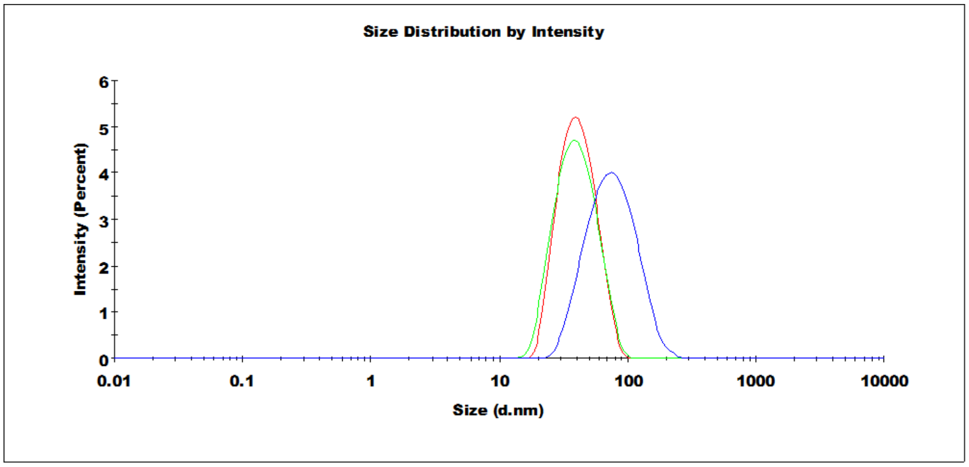

(B)

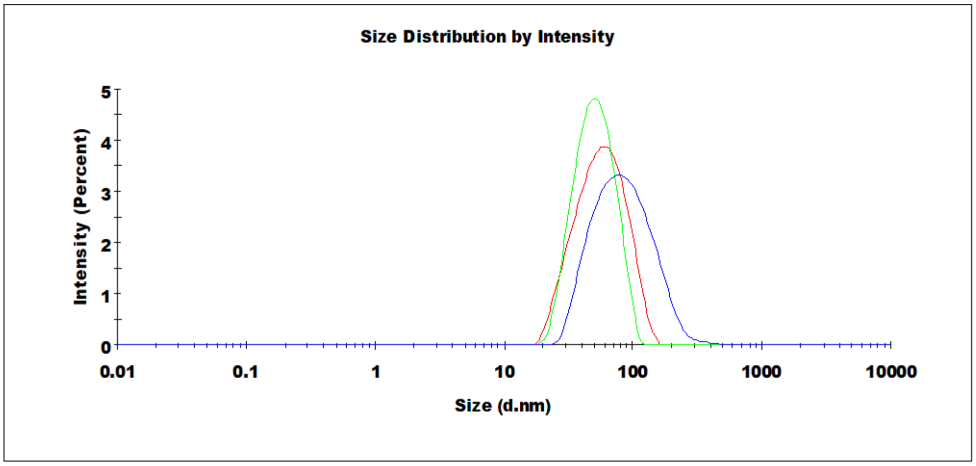

(C)

Figure S2. Size distribution by intensity for SA:BSA 0:1 (red line), SA:BSA 1:1 (green line), SA:BSA 10:1 (blue line) after (A) 24 hours at 277 K, (B) 20 hours at 338 K and (C) 48 hours at 338 K.

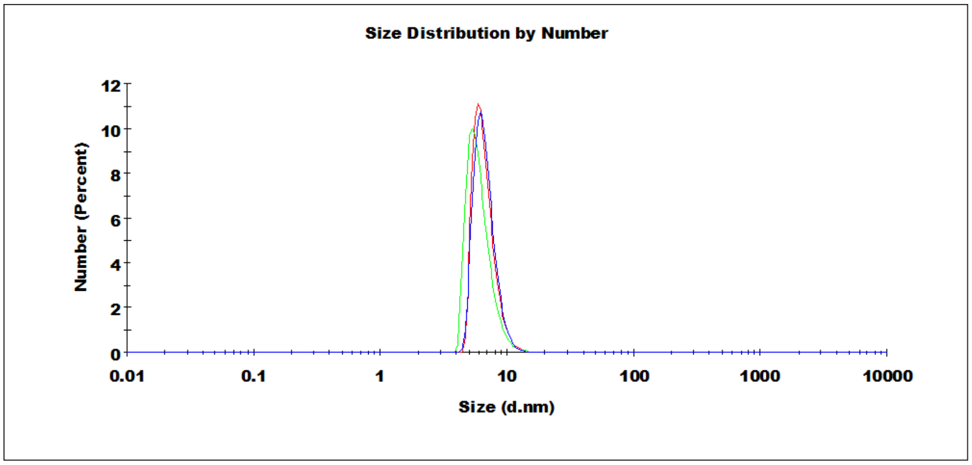

(A)

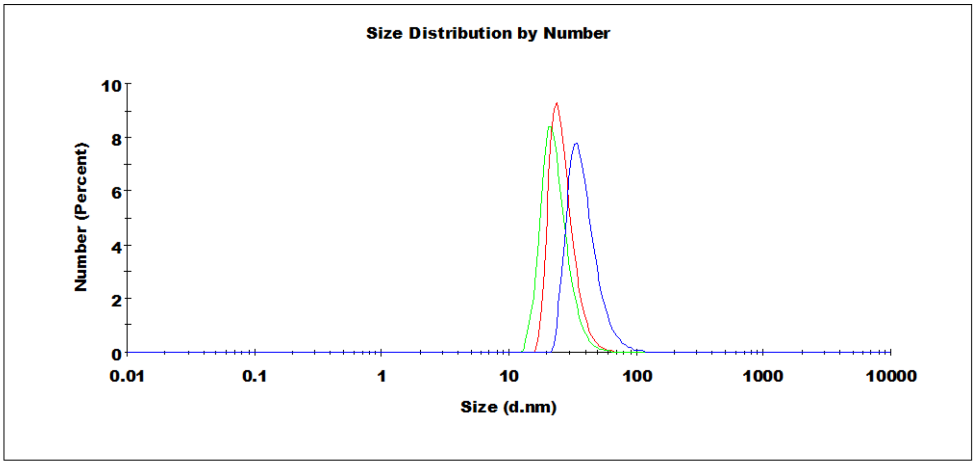

(B)

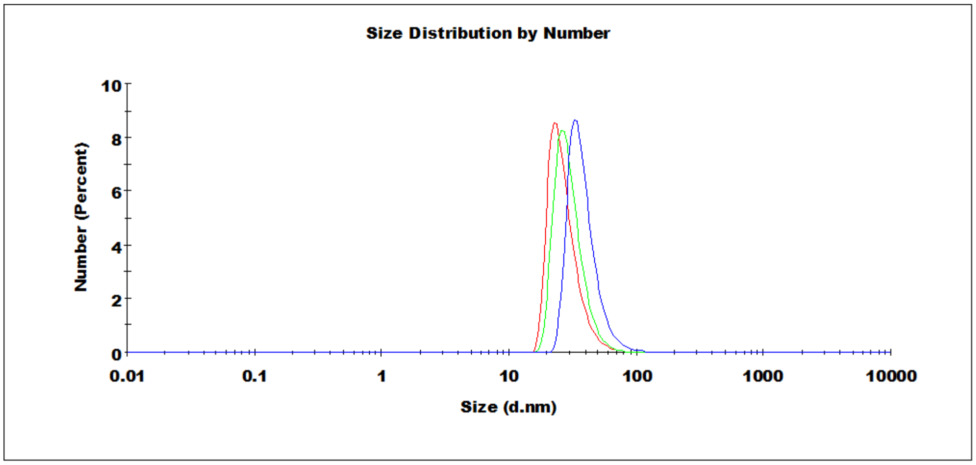

(C)

Figure S3. Size distribution by number for SA:BSA 0:1 (red line), SA:BSA 1:1 (green line), SA:BSA 10:1 (blue line) after (A) 24 hours at 277 K, (B) 20 hours at 338 K and (C) 48 hours at 338 K.

Table 1. The secondary structure content of protein in the absence and presence of SA evaluated using the K2D analysis algorithm from the Dichroweb website.

	system	helix	strand	random coil	NRMSD
	BSA	62	6	31	0.113
24 h at 277 K	SA:BSA 1:1	69	3	27	0.115
	SA:BSA 10:1	69	3	27	0.131
	BSA	55	10	35	0.145
20 h at 338 K	SA:BSA 1:1	58	8	34	0.156
	SA:BSA 10:1	58	8	34	0.155
	BSA	42	16	42	0.139
48 h at 338 K	SA:BSA 1:1	42	15	43	0.141
	SA:BSA 10:1	56	9	35	0.160
